# Modification of hybrid active bilayer for enhanced efficiency and stability in planar heterojunction colloidal quantum dot photovoltaics

**DOI:** 10.1186/1556-276X-8-488

**Published:** 2013-11-20

**Authors:** Seung Jin Heo, Seokhyun Yoon, Sang Hoon Oh, Hyun Jae Kim

**Affiliations:** 1 School of Electrical and Electronic Engineering, Yonsei University, Seoul 120-749, South Korea

**Keywords:** Hybrid active bilayer, Colloidal PbS quantum dot photovoltaics, Planar structure, Ambient air stability

## Abstract

Solution-processed planar heterojunction colloidal quantum dot photovoltaics with a hybrid active bilayer is demonstrated. A power conversion efficiency of 1.24% under simulated air mass 1.5 illumination conditions is reported. This was achieved through solid-state treatment with cetyltrimethylammonium bromide of PbS colloidal quantum dot solid films. That treatment was used to passivate Br atomic ligands as well as to engineer the interface within the hybrid active bilayer.

## Background

Much of the recent effort to develop photovoltaics (PV) has focused on third-generation PV. The third-generation PV is defined by cost and power conversion efficiency (PCE) greater than the Shockley-Queisser limit of 32% [1]. It can be reached through device architecture innovations, multiple-carrier generation using impact ionization, and new materials. Colloidal quantum dots (CQDs) have been proposed as useful materials for third-generation PV because of their ability to generate multiple excitons. Also, by changing the physical dimensions of CQDs, band gaps can be tuned from the visible to the infrared region using low-cost solution-processed fabrication. CQD PV has been studied in various ways using the following: Schottky CQD solar cells [2], depleted heterojunction CQD solar cells [3], and CQD-sensitized solar cells [4]. The highest PCE of CQD-based PV, 6%, has been achieved with depleted heterojunction CQD solar cells [5]; this PCE makes CQDs competitive with organic materials for the PV industry. CQD-based PV has lower cost per area and benefits from greater process flexibility compared with Si-based PV. However, some issues must still be overcome for PV applications. They are especially sensitive to humidity, light, and oxygen [6,7]. This sensitivity is the main cause of inferior charge transport, demanding a new strategy to solve these issues. Concurrent use of CQDs and organic compounds in devices has been one approach; these materials have typically been blended together [8-10]. To date, though, the PCE of a bilayer-based PV device has been much lower than that of blend-based PV because of poor morphology at the bilayer interface. In one example of a bilayer approach, Spoerke et al. reported that bilayer-based PV made with CdS CQDs and poly(3-hexylthiophene) (P3HT) had a PCE of 0.11% under simulated air mass (AM) 1.5 conditions [11].

Here, we introduce a planar heterojunction (PHJ) device architecture that has a ‘hybrid active bilayer,’ i.e., PbS CQD solid films layered with a blend of P3HT and [6,6]-phenyl-C61-butyric acid methyl ester (PCBM). This architecture offers broad absorption and efficient charge transport. Also, our study of the hybrid active bilayer clearly indicates its suitability as a new material for third-generation multijunction devices. Moreover, we have established an important dual role for solid-state treatment with cetyltrimethylammonium bromide (CTAB) used for atomic ligand passivation of PbS CQDs in a PHJ device. CTAB treatment serves to passivate the Br atomic ligands as well as engineer the interface within the hybrid active bilayer, leading to improved PCE and stability. We focused on the behavior of PbS CQDs to understand these phenomena.

## Methods

### Materials

Lead chloride (PbCl_2_, 98%), elemental sulfur, zinc acetate (Zn(Ac)_2_ · 2H_2_O), oleylamine (OLA, technical grade 70%), oleic acid (OA, technical grade 90%), 2-methoxyethanol, CTAB (99%), chlorobenzene (reagent, 99%), and toluene (anhydrous, 99.8%) were obtained from Sigma-Aldrich Corporation (St. Louis, MO, USA). Ethanol and methanol were purchased from Duksan Chemicals Co., Ltd. (Ansan-si, South Korea). P3HT and PCBM were purchased from Rieke Metals (Lincoln, NE, USA). All chemicals were used as received without further purification.

### Nanocrystal synthesis and device fabrication

A slurry of excess PbCl_2_ in OLA (1:2 molar ratio) was prepared at 100°C under a flow of N_2_. The temperature was increased to 120°C for 30 min. At the same time, elemental sulfur was dissolved in OLA (0.1:0.2 molar ratio) at 80°C over 30 min. The sulfur-OLA solution was added to the PbCl_2_-OLA slurry, and the temperature was raised to the growth temperature of 100°C and held there for 30 min. The mixture was then removed and quenched by pouring into cold toluene. OA was added to the PbS CQD suspension (20:3 volume ratio) at room temperature to exchange the OLA ligands for OA. The suspension was ultrasonicated and then centrifuged to remove the excess PbCl_2_. Ethanol was added to the retained supernatant to precipitate the quantum dots. The suspension was centrifuged, the supernatant was discarded, the precipitate was redispersed in toluene, and ethanol was added. The PbS CQDs containing OA ligands were isolated by centrifugation. Treatment with a methanol solution of CTAB was used to exchange OA ligands for the Br^-^ ones in the PbS CQD solid films using layer-by-layer spin coating. A three-step spin coating cycle was used: (1) 50 mg/mL of the PbS CQD solution was spin-coated, (2) 0.5 mL of the CTAB methanol solution was coated onto the PbS CQD solid films, and (3) the films were washed with methanol. Experiments were conducted at room temperature in air and without annealing during the ligand exchange process. This spin coating cycle was repeated seven times. OA-treated PbS CQD solid films, on the other hand, were made by simply spin coating PbS CQDs seven times, without using the other steps. Solution-processed ZnO thin films were spin-coated onto an indium tin oxide (ITO) substrate and annealed at 500°C for 4 h. The two types of PbS CQD solid films were then deposited. Chlorobenzene dispersions of P3HT and PCBM were spin-coated onto PbS CQD solid films in an argon-filled glove box and annealed at 120°C for 10 min. Layers of MoO_3_ (3 nm) and Au (100 nm) were deposited onto the active layer by thermal evaporation.

### Characterizations

The PbS CQDs were characterized by high-resolution transmission electron microscopy (HRTEM; Titan, FEI Co., Hillsboro, OR, USA). Current density-voltage characteristics were measured using an electrochemical analyzer (IviumStat, Ivium Technologies, Eindhoven, The Netherlands). An AM 1.5 solar simulator (Sun 2000, ABET Technologies, Milford, CT, USA) at 100 mW/cm^2^ intensity was used for illumination measurements. Absorption spectra were measured with a spectrophotometer (Cary 5G, Varian Inc., Palo Alto, CA, USA). This instrument was equipped with two light sources, i.e., a deuterium arc lamp and a quartz tungsten halogen lamp. X-ray photoelectron spectroscopy (XPS) spectra were measured using a commercial spectrometer (K-alpha, Thermo VG, Thermo Fisher Scientific, Waltham, MA, USA).

## Results and discussion

**Figure 1:**
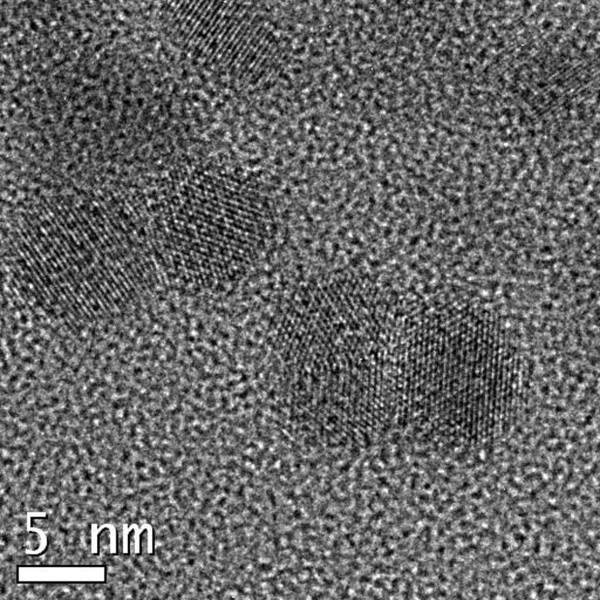
**HRTEM image of PbS CQDs.** The sample was applied to a TEM grid by evaporation at room temperature of a hexane solution.

Our synthesis was based on that of Hyeon [12]. The particle size and shape of our synthesized PbS CQDs were determined by HRTEM (Figure 1). The images revealed that the PbS CQDs were spherical, with an average size of about 5 nm. These PbS CQDs were passivated with oleylamine to prevent growth and oxidation in the colloidal solution.

**Figure 2:**
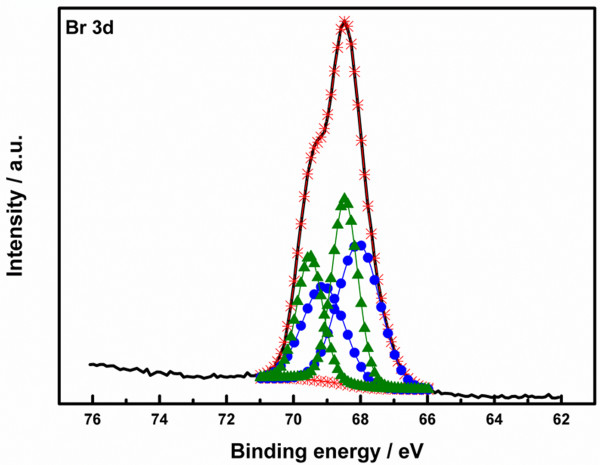
**XPS spectra of Br 3*d *core levels.** The dark curve is the original data and the orange asterisk is the superposition of fitted peaks. Peaks are indicated for bromine in unattached PbBr_2_ (blue circles) and bromine in PbBr_2_ (green triangles).

We used a solid-state treatment with CTAB for atomic ligand passivation [5]. This procedure exchanges OA for Br atomic ligands within a PbS CQD solid film. During this procedure, sufficient CTAB methanol solution was used to soak the PbS CQD solid films. XPS confirmed successful ligand exchange (Figure 2) [13]. We observed two chemical states which were halide and anion states; Sargent et al. [5] reported that only one state which was related to the binding of Br^-^ to Pb^2+^ existed. The difference of the chemical states of Br is caused by the amount of CTAB methanol solution applied to the PbS CQD solid films. From these results, we obtained not only the binding of Br^-^ to Pb^2+^, but also Br^-^ anions at the interface between each PbS CQD layer.

**Figure 3:**
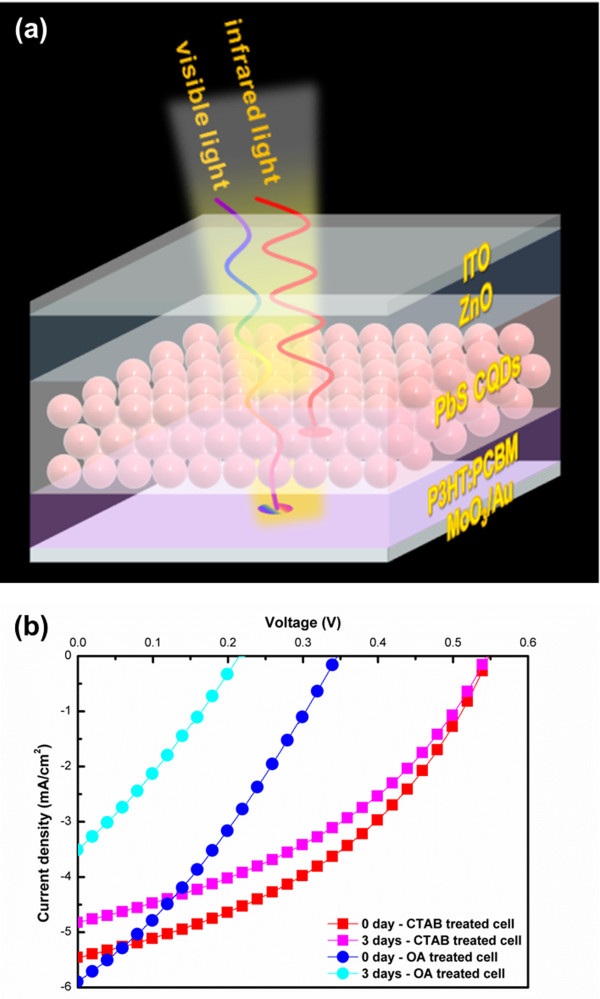
**Schematic drawing and current density-voltage characteristics. (a)** Schematic structure of the PHJ device. **(b)** Current density-voltage characteristics of the PHJ device with OA-treated PbS CQD solid films and CTAB-treated PbS CQD solid films.

A schematic drawing of our device and current density-voltage characteristics of PHJ devices on an ITO substrate (Au/MoO_3_/P3HT:PCBM/PbS/ZnO/ITO) are shown in Figure 3a, b. We prepared two types of PV to compare the effects of atomic and organic ligands: a CTAB-treated cell having PbS CQD solid films containing the Br atomic ligand and an OA-treated cell containing PbS CQD solid films containing OA ligands. The devices had similar structures (Figure 3a). Each device contained eight cells, each having an area of 4 mm^2^. To confirm ambient air stability, we kept the non-encapsulated devices in ambient air at room temperature for 3 days. Figure 3b and Table 1 detail the performance of the two types of devices. The CTAB-treated cell and OA-treated cell had almost the same short-circuit current density (*J*_SC_) value. However, the open-circuit voltage (*V*_OC_) of the CTAB-treated cell was one and a half times larger than the *V*_OC_ of the OA-treated cell. The CTAB-treated cell showed a twofold improvement in PCE (1.24% under AM 1.5 conditions), with *V*_OC_ = 0.55 V, *J*_SC_ = 5.41 mA/cm^2^, and fill factor (FF) = 42%. Also, the performance of the OA-treated cell after 3 days was much worse than that of the CTAB-treated cell; the CTAB-treated cell had almost constant *V*_OC_ and slightly lower *J*_SC_, whereas both *J*_SC_ and *V*_OC_ were lower for the OA-treated cell. Consequently, the PCE for the OA-treated cell had decreased by about 68%, whereas the PCE for the CTAB-treated cell had decreased only by about 15%. The decreased *J*_SC_ in the OA-treated cell was attributed to oxidation of the PbS CQD solid films over the 3-day exposure period. Solid-state treatment with CTAB forms a dense network within the CTAB-treated PbS CQD solid films which is not present in the OA-treated PbS CQD solid films. Oxygen penetrates relatively easily into OA-treated PbS CQD solid films. Moreover, voids from the OA ligand within the films act as trap sites.

**Table 1:** PHJ device performance

**Device**	***V***_**OC **_**(V)**	***J***_**SC **_**(mA/cm**^**2**^**)**	**FF (%)**	**PCE (%)**
CTAB-treated cell (0 day)	0.55	5.41	42	1.24
CTAB-treated cell (3 days)	0.54	4.78	41	1.06
OA-treated cell (0 day)	0.35	5.88	29	0.59
OA-treated cell (3 days)	0.21	3.47	26	0.19

**Figure 4:**
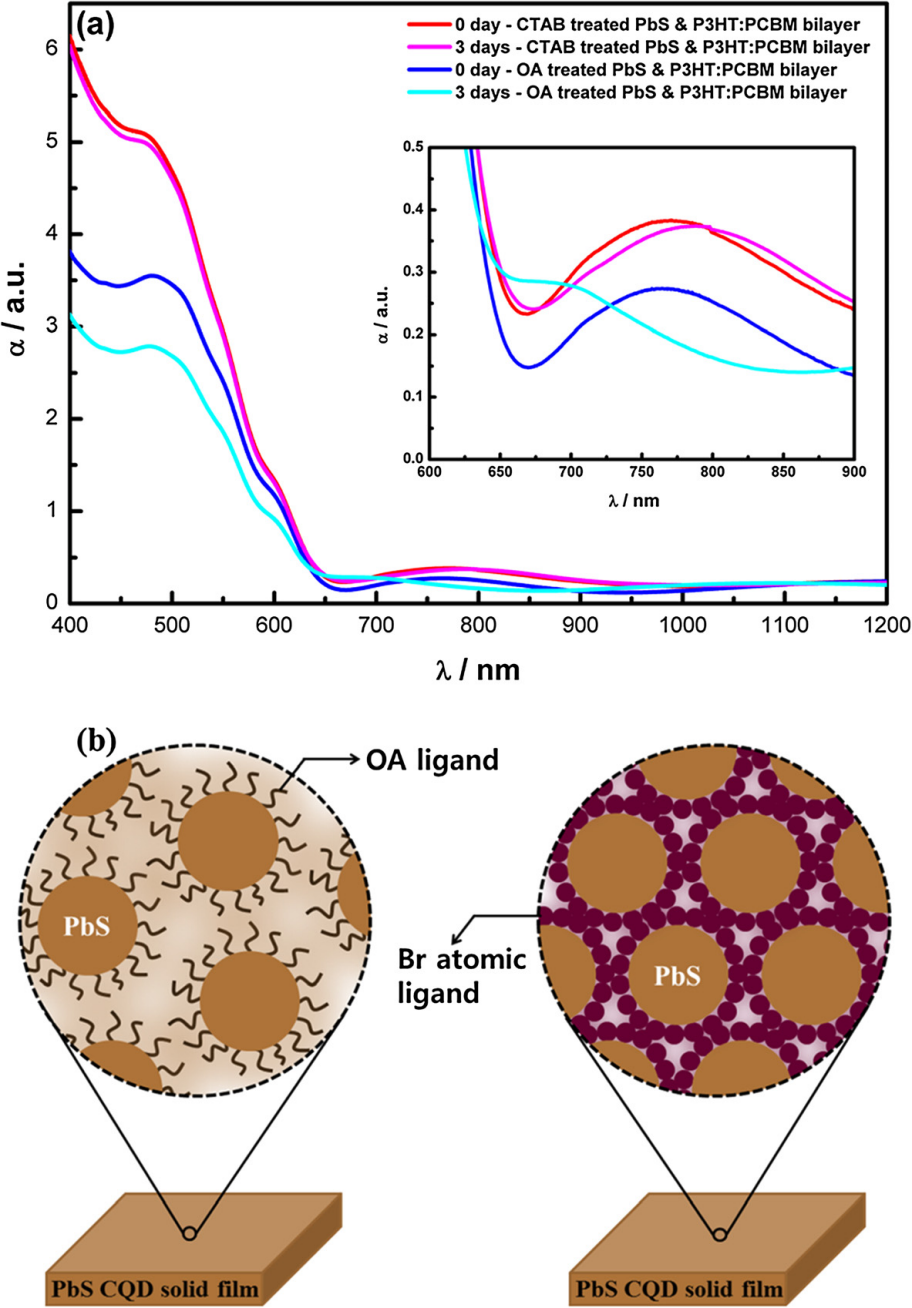
**Absorption spectra and schematic outline. (a)** Absorption spectra of hybrid active layers. **(b)** Schematic outline of the PbS CQD solid film. The left image represents the network in PbS CQD with OA ligand, and the right image represents the network in PbS CQD with Br atomic ligand.

We used grating spectrophotometry and XPS to determine the oxidation states of the various components. The first exciton peak related to PbS CQDs in the near-infrared region and interchain π-π^*^ absorption peaks related to P3HT in the visible region were observed in the optical absorption spectra (Figure 4a). Peaks for the CTAB-treated cells were red-shifted by 14.7 meV relative to those for the OA-treated cells. This shift was explained by the interdot spacing and a dipole layer within the hybrid active bilayer. For close-packed CQD solid films, red shifting of exciton peaks in optical absorption spectra often occurs because of interdot electronic couplings [14]. We can estimate the interdot distance in each PbS CQD solid film using the length of the ligands, i.e., a few angstroms in CTAB-treated PbS CQD solid films and a few nanometers in OA-treated PbS CQD solid films (Figure 4b). Also, excess bromine anions fully covering the PbS CQD solid films formed a dipole layer within the hybrid active bilayer. This dipole layer caused conduction-band energy-level alignment [15] and more efficient exciton dissociation. As a result, the *V*_OC_ of CTAB-treated cells was higher. Also, after 3 days, the first exciton peak of OA-treated cells broadened and shifted because of agglomeration and uneven oxidation within the films.

**Figure 5:**
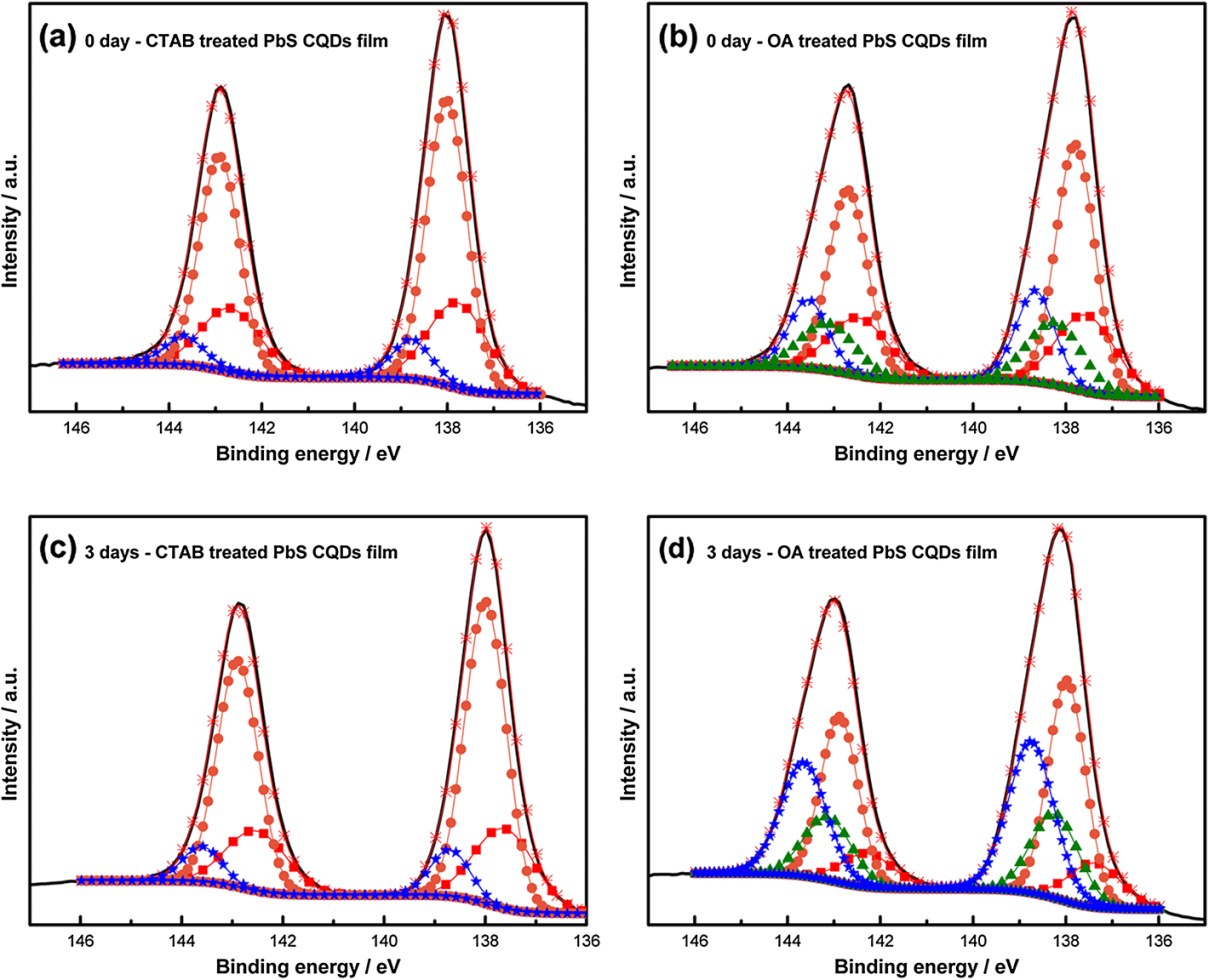
**XPS spectra of Pb 4*f *core levels to identify oxidized species. (a)** CTAB-treated PbS CQDs film (0 day), **(b)** OA-treated PbS CQDs film (0 day), **(c)** CTAB-treated PbS CQDs film (3 days), and **(d)** OA-treated PbS CQDs film (3 days). The dark curve is the original data and the orange asterisk is the superposition of fitted peaks. Peaks are indicated for elemental lead (red squares), lead in PbS (orange circles), lead in PbS linked to capping ligands (green triangles), and lead in PbSO_*x*_ (blue stars).

**Figure 6:**
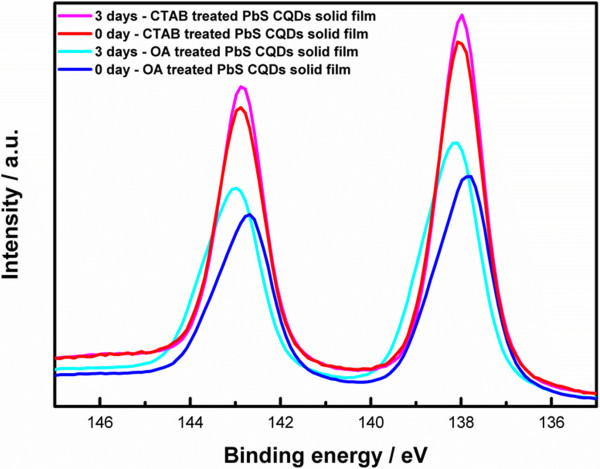
**XPS spectra of Pb 4** ***f *** **core levels.**

XPS was carried out over 3 days to study the changes in chemical states in PbS CQD solid films. The measurements were taken with monochromated Al Κα radiation at 1,486.6 eV with a 0° emission angle. The binding energy scale was calibrated using the C1s spectral component at 284.8 eV. As can be seen in Figure 5, we focused on the Pb 4*f* core level to identify oxidized species. A Shirley-type background was used. Each species was fitted to a Pb 4*f* doublet with an area ratio of 4:3 and a splitting energy of 4.9 eV [16]. Oxidized species were present in all samples because all samples were exposed to ambient air after synthesis. Air exposure, which formed oxidized species, occurred rapidly (within a few minutes after initial exposure) and continued for months [17]. The amount of oxidized species increased from 18% to 33% over 3 days for OA-treated PbS CQD solid films, whereas the amount remained stable at 10% for CTAB-treated PbS CQD solid films. Surface oxidation of PbS CQDs was also inferred from a shift from OA-treated PbS CQD solid films (Figure 6) [18]. These findings supported the current density-voltage characteristics. The highly oxidized species were the main cause of decreased *J*_SC_ and *V*_OC_ in OA-treated cell; this is because PbSO_*x*_ generates trap states below the conduction band [19].

## Conclusions

In conclusion, we have described an approach to improve *V*_OC_ and stability in a PHJ device using a hybrid active bilayer. The interface of this bilayer was modified by solid-state treatment with CTAB. The optimal CTAB-treated cell had a PCE of 1.24% under AM 1.5 conditions and maintained almost the same value (1.06%) over 3 days. Optical absorption spectra and XPS confirmed that Br atomic ligand passivation helped to prevent oxidation, while OA-treated PbS CQD solid films rapidly oxidized in ambient air at room temperature. A dipole layer between the PbS CQD layers formed as a consequence of the solid-state treatment with CTAB. For these reasons, the CTAB-treated cell had almost double the *V*_OC_ compared to the OA-treated cell. The possibility of using PbS CQDs as a multijunction with organic materials has been demonstrated in this study. We suggest that PbS CQDs be further explored as new materials for third-generation PV.

## Competing interests

The authors declare that they have no competing interests.

## Authors’ contributions

SJH and SY carried out the laboratory experiments. HJK and SHO participated in the discussion of the results, analyzed the data, and drafted the manuscript. All authors read and approved the final manuscript.
